# Regulation of Anti-Oxidative, Anti-Inflammatory, and Anti-Apoptotic Activity of Advanced Cooling Composition (ACC) in UVB-Irradiated Human HaCaT Keratinocytes

**DOI:** 10.3390/ijms21186527

**Published:** 2020-09-07

**Authors:** Jungha Park, Yong-Kyu Woo, Hyun-Jeong Cho

**Affiliations:** 1 Nature4 Co., Ltd., 110, Gyeongbuk Technopark, 27, Sampung-ro, Gyeongsan 38542, Korea; jh.park@nature4.co.kr; 2 Department of Biomedical Laboratory Science, College of Medical Science, Konyang University, 158, Gwanjeodong-ro, Daejeon 35365, Korea

**Keywords:** HaCaT keratinocytes, ACC, UVB, antioxidation, anti-inflammation, antiapoptosis

## Abstract

We recently demonstrated that advanced cooling composition (ACC) has effective ingredients that exhibit anti-inflammatory effects in RAW 264.7 cells stimulated with lipopolysaccharide (LPS) and exhibit strong antimicrobial effects on *Pseudomonas aeruginosa*, *Staphylococcus aureus*, MRSA (methicillin-resistant *Staphylococcus aureus*), *Candida albicans*, and *Streptococcus mutans*. To further investigate whether ACC has beneficial effects in ultraviolet B (UVB)-irradiated human keratinocytes (HaCaT cells), HaCaT cells were pretreated with ACC prior to UVB irradiation. Our data showed that ACC, which is effective at 100 µg/mL, is nontoxic and has an antioxidative effect against UVB-induced intracellular reactive oxygen species (ROS) in HaCaT cells. In addition, ACC exerts cytoprotective effects against UVB-induced cytotoxicity in HaCaT cells by inhibiting abnormal inflammation and apoptosis through the regulation of mitogen-activated protein kinase (MAPK) signals, such as jun-amino-terminal kinase (JNK), p38, and extracellular signal-regulated kinase (ERK). Therefore, these results indicate that ACC is a potentially beneficial raw material that possesses antioxidative, anti-inflammatory, and antiapoptotic effects against UVB-induced keratinocytes and may have applications in skin health.

## 1. Introduction

The skin is the largest organ in the body. In particular, the epidermis, the most superficial layer of the skin, serves to protect the body from the external environment and provides a barrier against the loss of internal fluids [1]. When the skin barrier function is damaged by various stimuli inside and outside the skin, the skin becomes dry or itches, and inflammatory responses occur [2]. In particular, keratinocytes make up the majority of epidermal cells, and their main function is to form keratin, induce cytokine secretion and, in severe cases, participate in various skin inflammatory responses.

Among the external stimuli that affect the skin, ultraviolet (UV) radiation, which is frequently encountered in everyday life, is a major environmental factor of skin damage. UV rays (100–400 nm wavelengths) consist of UVA (320–400 nm), UVB (280–320 nm), and UVC (200–280 nm) [3]. It is known that most skin damage caused by UV rays occurs with UVB (280–320 nm), and the wavelength of the UVB region is absorbed in the epidermal layer of the skin [4]. Accumulating evidence suggests that UVB irradiation not only induces nuclear DNA damage but also causes membrane destruction, resulting in cell loss or apoptosis [5,6]. It has been reported that oxidative stress on reactive oxygen species (ROS) produced by UV irradiation is a cause of skin inflammation and apoptosis [4,7]. As the ozone layer in the stratosphere around the planet is gradually depleted and the level of UV rays reaching the ground surface increases, the amount of UV radiation being exposed to the skin also increases. Eventually, acute or chronic UV irradiation is expected to cause skin disorders, including skin inflammation, burns, pigment diseases, aging, and skin cancer. The beneficial effects of natural products may play a key role in maintaining skin health, as well as preventing skin damage or inflammation. In particular, antioxidation, anti-inflammation, and antiapoptosis activities help to protect normal skin cells from UVB irradiation, and this study attempts to promote these activities through a mixture of herbal medicines.

We prepared the raw material by mixing 14 natural products at a specific ratio. Advanced cooling composition (ACC), mixed extract including *phellodendron* bark as the main ingredient, was invented after many years of compounding research to care damaged human skin without side effects, and it is obtained by vacuum low–temperature distillation–extraction. *Phellodendron* bark, which accounts for 24% of the ACC constituents, is a natural substance of the isoquinoline alkaloid series, which is dried bark of a *phellodendron* tree and mainly contains berberine as an indicator ingredient. Berberine has been reported to improve allergic inflammation and inhibit the expression levels of interleukin-6 (IL-6), chemokine (C-C motif) ligand 2 (CCL2), chemokine (C-C motif) ligand 7 (CCL7), and chemokine (C-X-C motif) ligand 8 (CXCL8), which are produced by the activation of eosinophils and are effective for atopy-like skin inflammation [8]. In addition, several studies have demonstrated that it has anti-inflammatory properties, and it inhibits the progression of oxidative stress, reduces cell death, and increases immunity [9,10,11]. Except for that, ACC consists of *Paeonia lactiflora* Pall., *Dictamnus dasycarpus* Turcz., *Inula helenium* etc., and their properties also include anti-inflammatory, anti-atopic, antimicrobial, and anti-allergic effects [12,13,14,15,16,17,18].

In our previous study, we demonstrated that ACC significantly attenuates the levels of nitric oxide (NO) production and pro-inflammatory cytokines, such as tumor necrosis factor-alpha (TNF-α), interleukin-1 beta (IL-1β), and interleukin-6 (IL-6), in RAW 264.7 murine macrophage cells stimulated with lipopolysaccharide (LPS). We also discovered that ACC has antimicrobial activities against several fungi [19]. However, whether ACC could show beneficial effects against UVB irradiation in human keratinocytes (HaCaT cells) has not been determined. Therefore, we examined whether ACC pretreatment could significantly change the level of ROS production and the mitogen-activated protein kinase (MAPK) signaling pathway and decrease the expression levels of factors associated with inflammation and apoptosis in UVB-irradiated HaCaT cells.

## 2. Materials and Methods

### 2.1. Preparation of ACC

**Figure 1:**
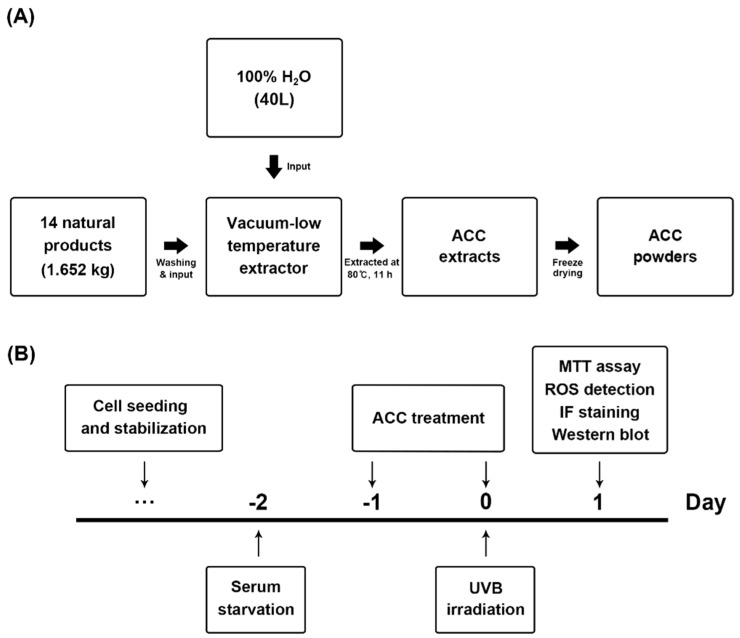
Process flow for extracting advanced cooling composition (ACC) and schematic diagram of the experimental design to determine whether ACC treatment affects heat shock-induced human keratinocytes (HaCaT) cells. (**A**) ACC extraction process. ACC is produced by the distilled extraction of 14 natural products, including *phellodendron* bark. (**B**) Timeline of ultraviolet B (UVB) irradiation and ACC treatment in HaCaT cells. HaCaT cells were treated with ACC starting 1 day before UVB irradiation and replaced daily until experiments were performed. After that, each HaCaT cell was evaluated by 3-(4,5-dimethyl-2-thiazolyl)-2,5-diphenyl-2H-tetrazolium bromide (MTT) assay, reactive oxygen species (ROS) detection, immunofluorescence staining, and Western blot analysis at 1 day after UVB irradiation.

A total of 14 different herbal medicines were selected through a literature search based on herbology, as previously described [19]. The herbal medicines using products packaged and sold as drugs were purchased from CK Pharm Co., Ltd. (128, Yangnyeongdong-gil, Dongdaemun-gu, Seoul, Korea) Fourteen natural products, including *Phellodendron* bark, *Scutellaria baicalensis*, *Paeonia lactiflora* Pall., *Dictamnus dasycarpus* Turcz., *Anemarrhena asphodeloides* Alumen, *Dryobalanops aromatica* Gaertner, *Mentha arvensis* var. *piperascens*, *Inula helenium*, *Syringa velutina* var. *kamibayashi*, *Corydalis incisa*, *Eclipta prostrata*, *Lonicera japonica*, and *Glycyrrhiza uralensis* were mixed according to their respective proportions (Table 1) and distilled for 11 h at 80 °C using 40 L of distilled water as a solvent (Figure 1A). To produce ACC powders, the supernatant was collected by filtration, and the water was removed using a freezing dryer. The ACC powders were prepared by being dissolved in Dulbecco’s phosphate-buffered saline (D-PBS; Welgene, Gyeongsan-si, Korea) according to each set concentration.

### 2.2. Constitutions Analysis with HPLC

A high-performance liquid chromatography (HPLC; Waters e2695 separations module, Waters Corporation, Milford, MA, USA) coupled with a 2998 photodiode array detector was performed for the qualitative determination of active compounds in the ACC. Briefly, ACC samples were injected using an autosampler at 4 °C (Waters 717 plus autosampler) and eluted through a Phenomenex Luna C18(2) column (4.6 × 250 mm × 5 μm; Phenomenex, Torrance, CA, USA) with a mobile phase of 0.1% trifluoroacetic acid in water/acetonitrile (90:10). The peaks of active compounds were analyzed and integrated using a Waters Empower^TM^ 3 software (Waters Corporation).

### 2.3. Cell Culture

The immortalized human keratinocytes, HaCaT cells, were purchased from AddexBio (San Diego, CA, USA). Cells were cultured in Dulbecco’s modified Eagle’s medium (DMEM; HyClone, GE Healthcare Life Sciences, Logan, UT, USA) supplemented with 10% heat-inactivated fetal bovine serum (FBS; HyClone) and antibiotics [100 U/mL penicillin and 100 µg/mL streptomycin (Invitrogen, Thermo Fisher Scientific, Waltham, MA, USA)] at 37 °C in a humidified 5% CO_2_ incubator. The HaCaT cells were cultured until they reached 90% confluence; then, they were subcultivated. Cells were starved for 1 day in serum-free media prior to being used for experiments. When required, HaCaT cells were treated with ACC in serum-free media and incubated for the same conditions.

### 2.4. UVB Irradiation

For UVB irradiation, HaCaT cells were cultured in 6-well plates (3 × 10^5^ cells/well), 12-well plates (1 × 10^5^ cells/well), and 96-well plates (1 × 10^4^ cells/well) for 1 day and were maintained in serum-free media for 1 day. When HaCaT cells were sufficiently adherent to the plates, the medium was removed, and the cells were washed twice with D-PBS (Welgene). Each well contained a thin layer of PBS to keep cell surfaces from drying. The lids of culture plates were opened, and the plates were placed in the UVP Crosslinker CL-1000M (Analytik jena, Jena, Germany), which emitted 302-nm radiation. Then, cells were irradiated with UVB at 40, 50, or 60 mJ/cm^2^ [UVB (40), UVB (50), or UVB (60)]. Irradiation doses were calculated using the following formula: Dose (mJ/cm^2^) = Exposure time (s) × Intensity (mW/cm^2^). After UVB irradiation, the cells were washed with warm PBS and incubated with serum-free DMEM for 1 day. When necessary, HaCaT cells were treated with ACC in serum-free media and were incubated under the same conditions.

### 2.5. Cell Viability Assay

Cytotoxicity induced by ACC treatment at various concentrations, UVB irradiation at various doses, and UVB irradiation in the presence of ACC in HaCaT cells was expressed as cell viability and measured using the 3-(4,5-dimethyl-2-thiazolyl)-2,5-diphenyl-2H-tetrazolium bromide (MTT) assay. HaCaT cells were cultured in 96-well plates (1 × 10^4^ cells/well) for 1 day in complete media and starved for 1 day in serum-free media. For ACC treatment, cells were treated with each concentration of ACC (0.001, 0.01, 0.1, 1, 10, 100, 250, 500, or 1000 µg/mL) in serum-free media and incubated for 1 day. As described above, UVB irradiation sections were irradiated with UVB (40), UVB (50), or UVB (60) and incubated for 1 day in serum-free media. To evaluate the effect of ACC following UVB irradiation in HaCaT cells, cells were pretreated with ACC before UVB irradiation, and after 1 day, cells were irradiated with the appropriate doses (40, 50, or 60 mJ/cm^2^). Subsequently, 20 µL of 3-(4,5-dimethyl-2-thiazolyl)-2,5-diphenyl-2H-tetrazolium bromide (MTT; Sigma-Aldrich, St. Louis, MO, USA) solution (5 mg/mL) was added, and the plate was incubated for an additional 4 h at 37 °C in a humidified 5% CO_2_ incubator. The supernatant was subsequently removed. Two hundred microliters of dimethyl sulfoxide (DMSO; Sigma-Aldrich) was added to solubilize a formazan product, and the optical density at 540 nm was measured using a SpectraMax iD3 plate reader (Molecular Devices, San Jose, CA, USA).

### 2.6. DPPH Radical Scavenging Assay

The 2,2-diphenyl-1-picrylhydrazyl (DPPH) radical scavenging activity of ACC was determined using the DPPH assay. Briefly, DPPH ethanol solution was added to several concentrations of ACC, ascorbic acid, quercetin, berberine, and baicalin (0.001, 0.01, 0.1, 1, 10, 100, 250, 500, or 1000 µg/mL) in 96-well plates. Ascorbic acid, quercetin, berberine, and baicalin were used as positive controls. After a 30-min incubation at room temperature (RT) in a dark room, the absorbance at 515 nm was measured using a SpectraMax iD3 plate reader (Molecular Devices). The DPPH radical scavenging activity of the sample was calculated by the following formula: DPPH radical scavenging activity (%) = (1 − As/Ab) × 100, where As is the absorbance of the sample and Ab is the absorbance of the blank.

### 2.7. Intracellular ROS Detection

The experiments were performed with two kinds of chemical fluorescence staining and fluorescence intensity measurement. Intracellular H_2_O_2_ levels were determined by measuring 2′,7′-dichlorodihydrofluorescein diacetate (DCFDA; Molecular Probes, Eugene, OR, USA), and O_2_^–^ levels were determined by measuring dihydroethidium (DHE; Molecular Probes). DCFDA is an H_2_O_2_-specific dye, and DHE has been used to monitor O_2_^–^ levels in cells [20]. To stain with reagent, the cells were seeded on round cover glass in 12-well plates (3 × 10^5^ cells/well) for 1 day and then maintained in serum-free media for 1 day. To measure the intensity of fluorescence, the cells were seeded in 96-well clear-bottom black plates (1 × 10^4^ cells/well) for 1 day and then maintained in serum-free media for 1 day. Then, the cells were stabilized in serum-free media without phenol red for at least 30 min before staining. To measure intracellular H_2_O_2_ and O_2_^–^, cells were incubated for 5 min at 37 °C with 20 μM DCFDA or DHE in the dark room. Next, cells were immediately observed using a Leica TCS SP8 (Leica microsystems, Wetlzer, Germany). Fluorescence was detected at 484/515 nm for DCFDA staining and at 485/585 nm for DHE staining. Fluorescence intensity was measured using a SpectraMax iD3 plate reader (Molecular Devices), and values were expressed with respect to the control.

### 2.8. Immunofluorescence Staining Procedure

HaCaT cells grown on round cover glass in the 12-well plates (3 × 10^5^ cells/well) were treated as indicated above and fixed in ice-cold 100% methanol for 10 min. Cells were permeabilized for 10 min at RT with D-PBS containing 1% bovine serum albumin (BSA) and 0.1% Triton X-100, and cells were subsequently blocked in D-PBS containing 1% BSA at RT for 1 h. Then, the cells were incubated for 1 day with one of the following primary antibodies: mouse anti-TNFα (1:500; Santa Cruz Biotechnology, Santa Cruz, CA, USA), mouse anti-IL-1β (1:500; Santa Cruz Biotechnology), rabbit anti-cleaved caspase-3 (c-caspase 3; 1:400; Cell Signaling, Beverly, MA, USA), mouse anti-cleaved-poly (ADP-ribose) polymerase (c-PARP; 1:400; Cell Signaling), rabbit anti-phospho-p38 MAPK (p-p38; 1:1600; Cell Signaling), and rabbit anti-phospho-p44/42 MAPK (p-ERK1/2; 1:200; Cell Signaling). After 1 day, cells were rinsed and incubated with appropriate secondary antibodies for 1 h, and cover glasses were subsequently washed and mounted onto microscope slides using ProLong™ Gold Antifade Mountant with 4′,6-diamidino-2-phenylindole (DAPI; Invitrogen). Fluorescence images were acquired using Leica TCS SP8 (Leica Microsystems, Wetzlar, Germany) and analyzed with Leica Application Suite X (LAS X).

### 2.9. Western Blot Analysis

Whole cell lysates in HaCaT cells were prepared with protease inhibitor- and phosphate inhibitor-containing radioimmunoprecipitation assay (RIPA) lysis buffer [50 mM Tris-HCl (pH 7.5), 150 mM NaCl, 1% Triton X-100, 1% sodium deoxycholate, 0.1% SDS and 2 mM ethylenediaminetetraacetic acid (EDTA)] and centrifuged at 4 °C for 15 min at 14,000 g. The supernatant was transferred to a fresh tube, and the concentration was determined using a bicinchoninic acid assay (BCA) kit (Bio-Rad Laboratories, Hercules, CA, USA). The samples were boiled at 95 °C for 5 min before gel loading, and equal amounts of protein were loaded into each lane with the loading buffer. Proteins were separated using sodium dodecyl sulfate (SDS)-polyacrylamide gel electrophoresis (PAGE; Bio-Rad Laboratories) and electrophoretically transferred to polyvinylidene difluoride membranes (PVDF; Millipore, Darmstadt, Germany). The membranes were subsequently incubated overnight at 4 °C with specific primary antibodies with 5% skim milk or 5% BSA in tris-buffered saline with tween-20 (TBS-T) solution: mouse anti-TNFα (1:500; Santa Cruz Biotechnology), mouse anti-IL-1β (1:500; Santa Cruz Biotechnology), rabbit anti-cyclooxygenase 2 (COX-2; 1:1000; Cell Signaling), rabbit anti-inducible nitric oxide synthases (iNOS; 1:500; Abcam, Cambridge, MA, USA), rabbit antiapoptosis signal-regulating kinase 1 (ASK1; 1:1000; Cell Signaling), rabbit anti-phospho-ASK1 (p-ASK1; 1:1000; Cell Signaling), rabbit anti-stress-activated protein kinase/jun-amino-terminal kinase (SAPK/JNK; 1:1000; Cell Signaling), rabbit anti-phospho-SAPK/JNK (p-SAPK/JNK; 1:1000; Cell Signaling), rabbit anti-p38 MAPK (p38; 1:1000; Cell Signaling), rabbit anti-phospho-p38 MAPK (Thr180/Tyr182) (p-p38; 1:1000; Cell Signaling), rabbit anti-p44/42 MAPK (ERK1/2; 1:1000; Cell Signaling), rabbit anti-p-ERK1/2 (1:1000; Cell Signaling), rabbit anti-bax (1:1000; Cell Signaling), mouse anti-caspase-9 (1:1000; Cell Signaling), rabbit anti-cleaved-caspase-9 (c-caspase-9; 1:1000; Cell Signaling), rabbit anti-caspase-3 (1:1000; Cell Signaling), rabbit anti-c-caspase-3 (1:1000; Cell Signaling), mouse anti-caspase-8 (1:1000; Cell Signaling), rabbit anti-cleaved-caspase-8 (c-caspase-8; 1:1000; Cell Signaling), rabbit anti-PARP (1:1000; Cell Signaling), mouse anti-c-PARP (1:1000; Cell Signaling), and rabbit anti-β-actin (1:1000; Cell Signaling). After washing with TBS-T, the membranes were incubated with the appropriate secondary antibodies (Invitrogen) for 1 hour at RT, and the blots were finally developed using enhanced chemiluminescence (ECL) Western blot detection reagents (GE Healthcare Life Sciences, Logan, UT, USA). Target protein bands were observed and photographed by a luminescent image analyzer, ImageQuant LAS 500 (GE Healthcare Life Sciences). For semiquantitative analyses, the density of the protein bands was measured using a computer imaging device and accompanying software (Fuji Film, Tokyo, Japan).

### 2.10. Statistical Analysis

All values are expressed as the mean ± standard error of the mean (SEM). All experiments were repeated at least three times. Multiple comparisons among the groups were performed by one-way analysis of variance (ANOVA) followed by Tukey’s post hoc test. *p*-values < 0.05 were considered to be significant. All statistical analyses were performed using SigmaPlot 12.0 (Systat Software, San Leandro, CA, USA).

## 3. Results

### 3.1. Identification of Active Compounds from ACC

**Figure 2:**
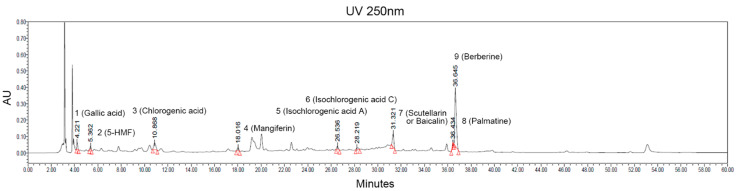
HPLC–UVλ=250nm chromatograms of ACC extract produced from 14 different herbal medicines. The quantification of samples was preformed using a Waters e2695 separation module, 2998 photodiode array detector, and column used the Phenomenex Luna C18(2), 250 × 4.6 mm, 5 μm, maintained at ambient room temperature. Two special symbols marked on each peak indicate the beginning and end of the candidate signal. 1: gallic acid, 2: 5-hydroxymethylfurfural (5-HMF), 3: chlorogenic acid, 4: mangiferin, 5: isochlorogenic acid A, 6: isochlorogenic acid C, 7: scutellarin or baicalin, 8: palmatine, 9: berberine.

The ACC stock solution obtained by vacuum low–temperature distillation–extraction was filtered and sterilized, and then, it was applied to subsequent experiments for the quantitative analysis of active compounds constituting ACC. A high-performance liquid chromatographic method coupled with UV was conducted for qualitative determination of the compounds in the ACC. The main compounds contained in ACC were identified by comparing the retention time with the reference standard as follows: gallic acid, 5-hydroxymethylfurfural (5-HMF), chlorogenic acid, mangiferin, isochlorogenic acid A, isochlorogenic acid C, scutellarin or baicalin, palmatine, and berberine (Figure 2).

### 3.2. Cell Viability after UVB Irradiation in ACC-Treated HaCaT Cells

**Figure 3:**
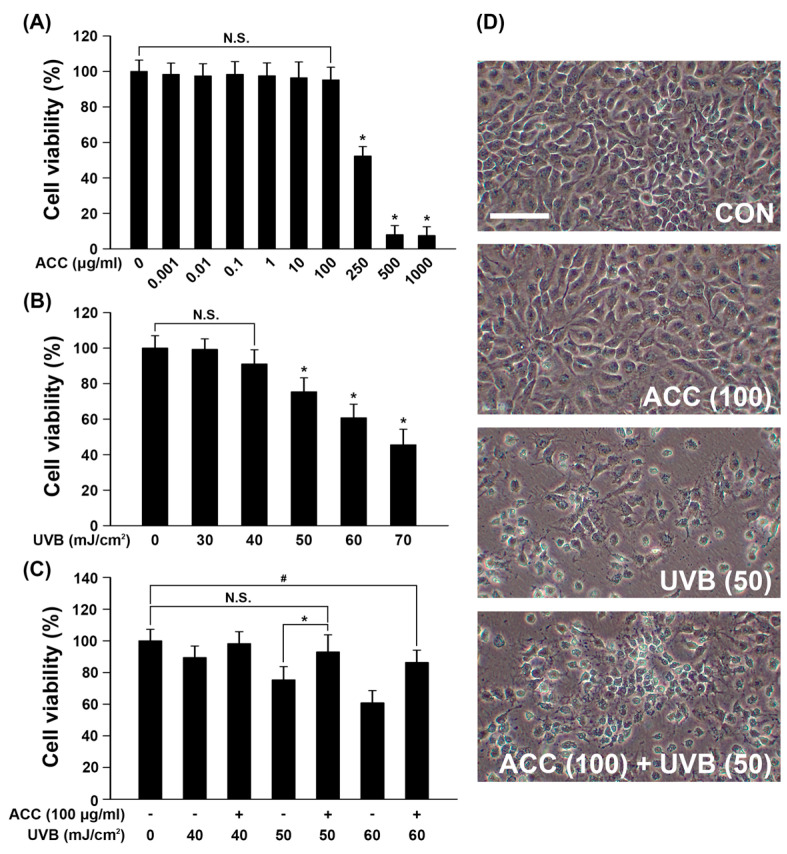
Viability of HaCaT cells under various conditions. (**A**) HaCaT cell viability on various concentrations of ACC. Cell viability was measured by the MTT assay at 1 day after ACC treatment (0, 0.001, 0.01, 0.1, 1, 10, 100, 250, 500, or 1000 μg/mL). * *p* < 0.001 vs. no treatment. N.S., not significant. (**B**) HaCaT cell viability after treatment with various concentrations of UVB. Cell viability was measured by the MTT assay at 1 day after 0, 30, 40, 50, or 60 mJ/cm^2^ UVB irradiation. * *p* < 0.001 vs. no treatment. N.S., not significant. (**C**) HaCaT cell viability after ACC treatment with UVB irradiation. Cell viability was measured by the MTT assay at 1 day after 40, 50, or 60 mJ/cm^2^ UVB irradiation in HaCaT cells pretreated with 100 µg/mL ACC. * *p* < 0.001, UVB (50) vs. ACC (100) + UVB (50); # *p* = 0.002, no treatment vs. ACC (100) + UVB (60). N.S., not significant. (**D**) Representative figures of cell morphology at CON, ACC (100) alone, UVB (50), and ACC (100) + UVB (50) in HaCaT cells. CON, control cells. Scale bar, 400 μm. All experiments were repeated at least three times, and similar results were obtained.

In keeping with the process for ACC extraction and the designed experimental schedules (Figure 1A,B), we evaluated the beneficial effects of ACC on UVB irradiation in HaCaT cells. First, we investigated whether ACC includes cell protective properties against UVB irradiation, and the effect of ACC on cell viability was assessed in HaCaT cells. In other words, we evaluated the effect of ACC concentration and UVB irradiation on cell viability under several conditions. Based on the screened conditions, we measured the cell viability when UVB was irradiated to ACC-treated HaCaT cells. Our results showed that cell viability was significantly decreased at 2 days after treatment with ACC concentrations above 250 µg/mL [ACC (250)] compared with no treatment. When quantified and expressed as the percentage of cell viability after ACC (250) treatment compared with no treatment, ACC (250) decreased the number by 52% compared with no treatment (Figure 3A). Next, cell viability after various doses of UVB was evaluated in HaCaT cells. The results showed that significant cytotoxicity was observed at 1 day after UVB irradiation at doses above 50 mJ/cm^2^ in HaCaT cells. When quantified and expressed as a percentage of cell viability after UVB (50) treatment compared with no treatment, UVB (50) decreased the number by 25% compared with no treatment (Figure 3B). However, UVB (30) and UVB (40) treatment caused no significant decrease in cell viability. Accordingly, ACC (100), which is a limit concentration that does not show cellular toxicity in HaCaT cells, was set as a reference concentration, and the effect of ACC on UVB irradiation was measured through cell viability. The results showed that ACC pretreatment significantly increased the cell viability of UVB (50) in HaCaT cells (Figure 3C). Similar to the cell viability results, the images showed morphological differences in HaCaT cells between UVB (50) treatment and ACC (100) plus UVB (50) treatment of cells (Figure 3D). These results suggest that ACC has protective potential against UVB irradiation.

### 3.3. Attenuation of UVB-Induced Intracellular ROS in ACC-Treated HaCaT Cells

**Figure 4:**
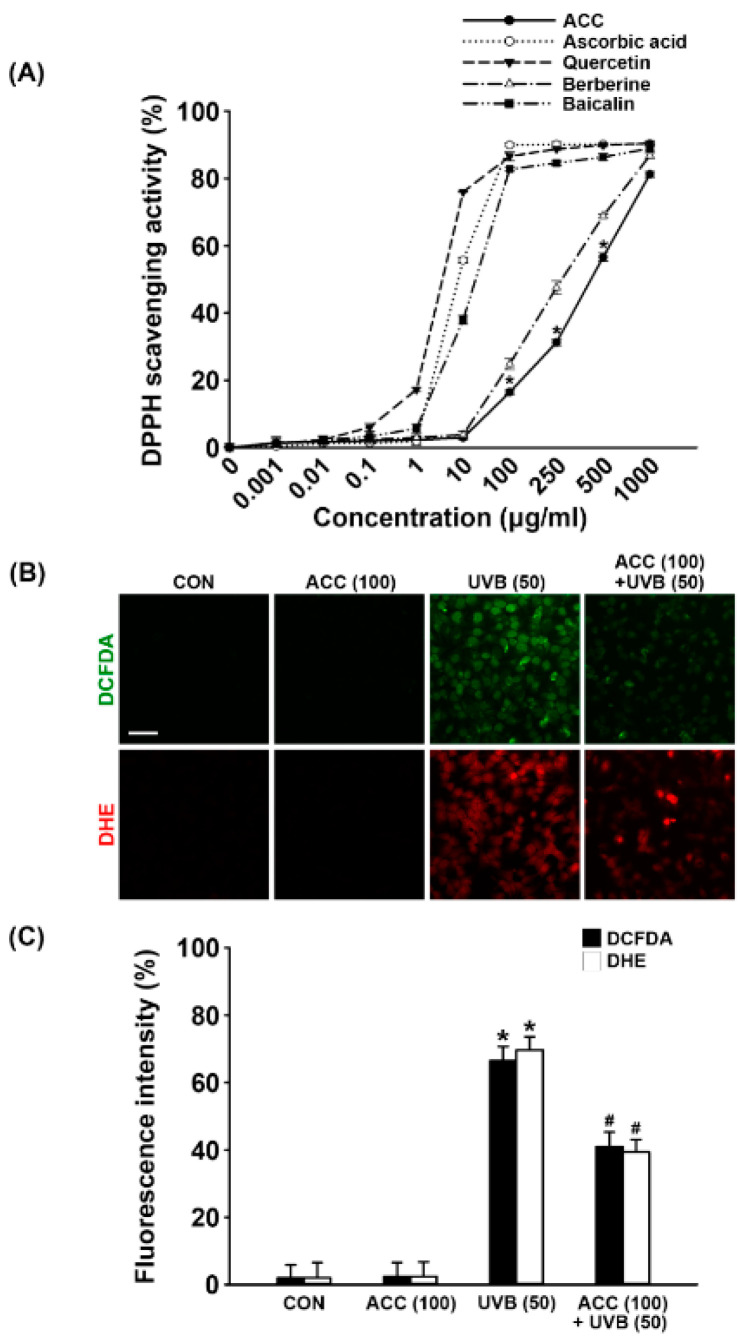
Antioxidative effect of ACC in UVB-irradiated HaCaT cells. (**A**) Effect of ACC and single natural compounds on 2,2-diphenyl-1-picrylhydrazyl (DPPH) radical scavenging activity. Four active compounds, ascorbic acid, quercetin, berberine, and baicalin, were used as positive controls. * *p* < 0.001 vs. no treatment. (**B**) Images of 2′,7′-dichlorodihydrofluorescein diacetate (DCFDA) and dihydroethidium (DHE) on ACC treatment in UVB-irradiated cells. DCFDA and DHE stained for H_2_O_2_ (**green**) and O_2_^–^ (**red**) in HaCaT cells following UVB irradiation at 50 mJ/cm^2^. (**C**) The fluorescence intensity of DCFDA and DHE on ACC treatment in UVB-irradiated cells. CON, control cells. Scale bar, 50 μm. * *p* < 0.001 vs. CON; # *p* < 0.001 vs. UVB (50). All experiments were repeated at least three times, and similar results were obtained.

ACC is a raw material extracted from a mixture of 14 natural products in a specific ratio, and ACC is expected have antioxidative effects due to its natural properties. Therefore, before the application to HaCaT cells, a DPPH assay was performed to measure the antioxidative effect of ACC itself. The DPPH free radical scavenging assay is an experimental method for measuring the reducing power of a sample. The free radical scavenging ability of the sample is determined by donating electrons to the free radical of DPPH. After measuring the antioxidative effect of ACC, a significant free radical scavenging activity was observed: 16.48% at the concentration of 100 µg/mL, 31.24% at 250 µg/mL, 56.64% at 500 µg/mL, and 81.21% at 1000 µg/mL (Figure 4A). The DPPH scavenging activity of ACC is similar to the activity of berberine, which is reasonable considering that the main ingredient of *Phellodendron* bark, a representative substance of ACC, is berberine. Next, we evaluated whether ACC could scavenge UVB-induced intracellular ROS, such as H_2_O_2_ and O_2_^–^. As demonstrated by ROS fluorescence staining, our results showed that there were apparent increases in H_2_O_2_ (green; DCFDA) and O_2_^-^ (red; DHE) in HaCaT cells at 1 day after UVB (50) irradiation compared with control cells (Figure 4B). Consistent with the ROS fluorescent staining results, fluorescence intensity also exhibited increases in intracellular ROS, such as H_2_O_2_ and O_2_^–^, at 1 day after UVB (50)-irradiated HaCaT cells. However, ACC pretreatment significantly reduced the production of H_2_O_2_ and O_2_^–^ at 1 day after UVB (50) irradiation in HaCaT cells (Figure 4B,C). Although ACC is not as efficacious as a single compound with excellent antioxidative efficacy, these results suggest that ACC contains components with significant antioxidative activity.

### 3.4. Effect of ACC on the Downregulation of the Pro-Inflammatory Cytokines, COX-2, and iNOS in UVB-Irradiated HaCaT Cells

**Figure 5:**
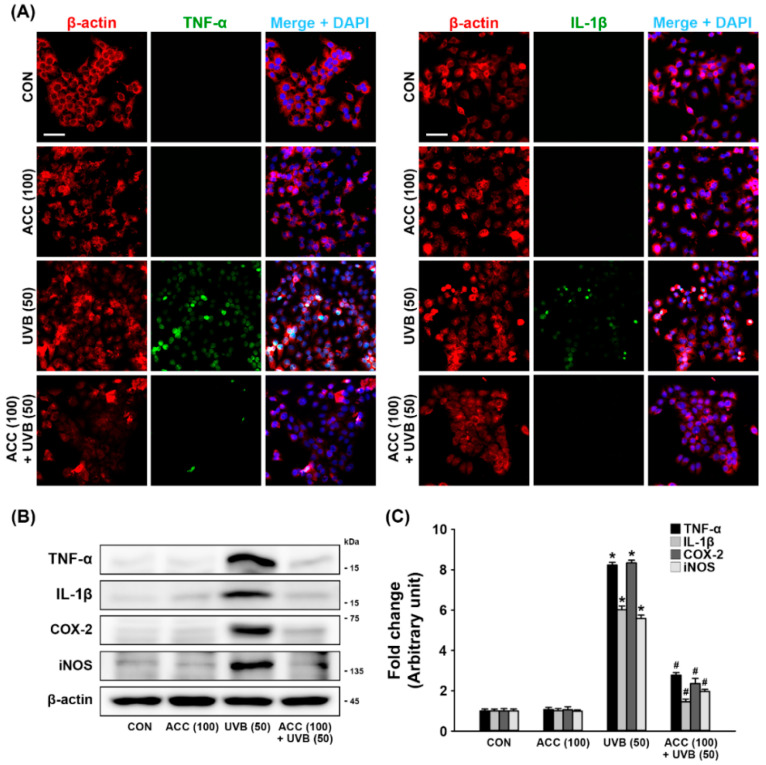
Anti-inflammatory effect of ACC in UVB-irradiated HaCaT cells. (**A**) Representative images of immunofluorescence labeling for β-actin (**red**) and tumor necrosis factor-alpha (TNF-α) (**green**) or β-actin and IL-1β (**green**) in UVB-irradiated (50 mJ/cm^2^) HaCaT cells. Scale bar, 50 μm. (**B**,**C**) Effects of TNF-α, interleukin-1 beta (IL-1β), cyclooxygenase 2 (COX-2), and inducible nitric oxide synthases (iNOS) in UVB-irradiated (50 mJ/cm^2^) HaCaT cells. TNF-α, IL-1β, COX-2, and iNOS were measured at 1 day after UVB in HaCaT cells. * *p* < 0.001 vs. CON; # *p* < 0.001 vs. UVB (50). All experiments were repeated at least three times, and similar results were obtained.

To examine whether ACC could inhibit the UVB-induced inflammatory responses in HaCaT cells, double immunofluorescence staining and Western blot analysis were performed to evaluate the protein levels of pro-inflammatory cytokines, such as TNF-α and IL-1β. As demonstrated in the double immunofluorescence labeling, our results showed that ACC treatment did not induce any changes in the normal state of TNF-α and IL-1β, and there were distinct increases in TNF-α and IL-1β in HaCaT cells at 1 day after UVB (50) irradiation compared with control cells (Figure 5A). Consistent with the immunofluorescence results, Western blot analysis also showed significant increases in pro-inflammatory cytokines, such as TNF-α and IL-1β, in HaCaT cells at 1 day after UVB (50) irradiation compared with control cells (Figure 5B,C). In addition, the protein levels of COX-2 and iNOS, which are factors that play a role in inflammation and carcinogenesis, were also measured by Western blot analysis, and the results showed a significant increase following UVB (50) irradiation in HaCaT cells (Figure 5B,C). However, ACC pretreatment significantly attenuated the production of TNF-α, IL-1β, COX-2, and iNOS following UVB (50) irradiation in HaCaT cells (Figure 5B,C). These results suggest that ACC-treated keratinocytes could maintain a basal level of the pro-inflammatory cytokines, COX-2, and iNOS against UVB irradiation, indicating that ACC can play a role in controlling inflammatory responses.

### 3.5. Regulation of JNK/p38/ERK Signaling Pathways by ACC Pretreatment

**Figure 6:**
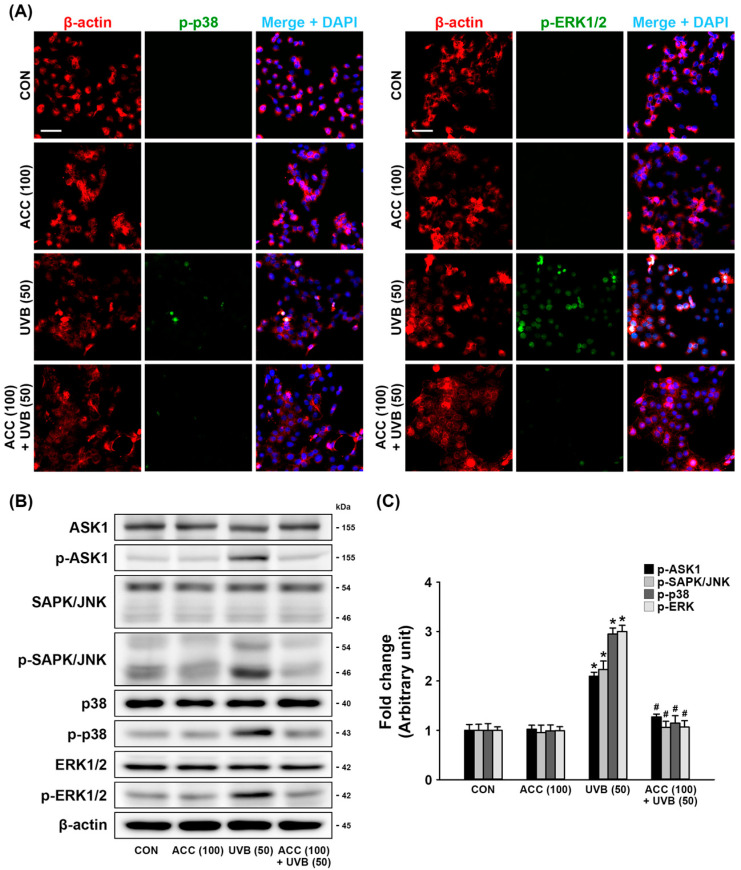
Effect of ACC treatment on mitogen-activated protein kinase (MAPK) expression in UVB-irradiated HaCaT cells. (**A**) Representative images of immunofluorescence labeling for β-actin (red) and phospho-p38 (p-p38; green) or β-actin and phospho-p44/42 MAPK (p-ERK1/2; green) in UVB-irradiated (50 mJ/cm^2^) HaCaT cells. Scale bar, 50 μm. (**B**,**C**) Effects of antiapoptosis signal-regulating kinase 1 (ASK1), phospho-ASK1 (p-ASK1), stress-activated protein kinase/jun-amino-terminal kinase (SAPK/JNK), phospho-SAPK/JNK (p-SAPK/JNK), p38, p-p38, extracellular signal-regulated kinase 1/2 (ERK1/2), and p-ERK1/2 in UVB-irradiated (50 mJ/cm^2^) HaCaT cells. ASK1, p-ASK1, SAPK/JNK, p-SAPK/JNK, p38, p-p38, ERK1/2, and p-ERK1/2 were measured 1 day after UVB in HaCaT cells 1 day after UVB irradiation. * *p* < 0.001 vs. CON; # *p* < 0.001 vs. UVB. All experiments were repeated at least three times, and similar results were obtained.

JNK, p38, and ERK are strongly correlated with acute inflammation [21]. Therefore, we investigated whether ACC pretreatment could downregulate the aberrant phosphorylation of SAPK/JNK, p38, or ERK1/2 in UVB-irradiated HaCaT cells, and the levels of biomarkers associated with their signaling pathway were measured by double immunofluorescence staining and Western blot analysis. As demonstrated by double immunofluorescence labeling, UVB (50) irradiation aberrantly upregulated the expression levels of p-p38 and p-ERK1/2 in HaCaT cells compared with control cells, while increased expression was significantly alleviated to levels of the ground state in ACC-treated HaCaT cells (Figure 6A). Consistent with the immunofluorescence staining results, Western blot analysis also showed that increased expression levels of p-ASK1, p-SAPK/JNK, p-p38, and p-ERK1/2 in UVB (50)-irradiated HaCaT cells (Figure 6B,C) were significantly decreased by ACC pretreatment compared with UVB (50)-irradiated HaCaT cells (Figure 6B,C). These results indicate that UVB irradiation in HaCaT cells could upregulate the JNK/p38/ERK signaling pathways. However, the inhibitory effect of ACC on UVB-induced MAPK phosphorylation suggests that it might result in blockage of the inflammatory responses associated with pro-inflammatory cytokine production and COX-2 expression.

### 3.6. Inhibition Effect of ACC in UVB-Induced Pro-Apoptotic Molecules

**Figure 7:**
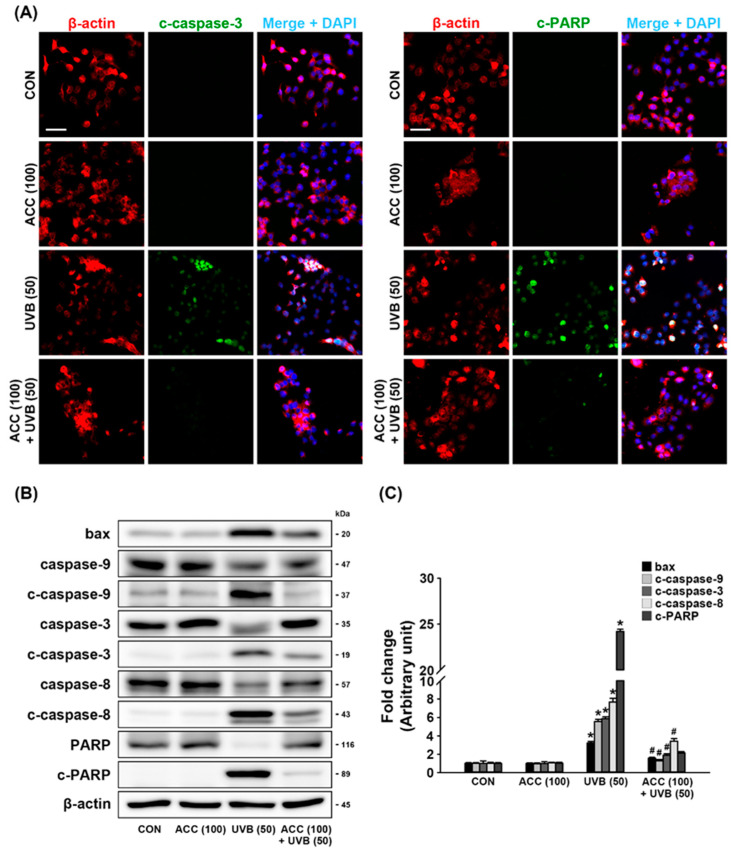
Antiapoptotic effect of ACC in UVB-irrdiated HaCaT cells. (**A**) Representative images of immunofluorescence labeling for β-actin (red) and cleaved-caspase-3 (c-caspase-3; green) or β-actin and cleaved-poly (ADP-ribose) polymerase (c-PARP; green) in UVB-irradiated (50 mJ/cm^2^) HaCaT cells. Scale bar, 50 μm. (**B**,**C**) Effects of bax, caspase-9, c-caspase-9, caspase-3, c-caspase-3, caspase-8, c-caspase-8, PARP, and c-PARP in UVB-irradiated (50 mJ/cm^2^) HaCaT cells. Bax, caspase-9, c-caspase-9, caspase-3, c-caspase-3, caspase-8, c-caspase-8, PARP, and c-PARP were measured at 1 day after UVB irradiation of HaCaT cells. * *p* < 0.001 vs. CON; # *p* < 0.001 vs. UVB. All experiments were repeated at least three times, and similar results were obtained.

We evaluated whether ACC pretreatment attenuates UVB-induced pro-apoptotic molecules. The expression levels of pro-apoptotic molecules were measured by double-immunofluorescence staining and Western blot analysis. As demonstrated by double immunofluorescence labeling, UVB (50) irradiation increased the expression levels of c-caspase-3 and c-PARP in HaCaT cells compared with control cells. However, this upregulation was significantly decreased in ACC-treated HaCaT cells (Figure 7A). Consistent with the immunofluorescence staining results, Western blot analysis also showed that increased expression levels of bax, c-caspase-9, c-caspase-3, c-caspase-8, and c-PARP in UVB (50)-irradiated HaCaT cells (Figure 7B) were significantly reduced by pretreatment with ACC compared with UVB(50)-irradiated HaCaT cells (Figure 7B,C). These results suggest that UVB (50) irradiation in HaCaT cells can activate a part of the pro-apoptotic signaling cascades, and ACC has an effective ingredient, including antiapoptotic effects.

## 4. Discussion

Keratinocytes make up 95% of the epidermal mass, and they play an important role in skin physiology through autocrine or paracrine effects between themselves [22]. The continued production of specific intracellular signaling factors is not observed in the basal state, but various stimuli both inside and outside the cell, including endotoxins or UV, can trigger the expression of pro-inflammatory cytokines or pro-apoptotic signaling molecules [23]. UVB is a major environmental factor of skin damage, and as mentioned in the Introduction, it can reach the epidermal basal cell layer of the skin, meaning that the effect is noticeable in epidermal cells that contain keratinocytes. For example, exposure to solar UVB radiation (280–320 nm) can cause a number of disorders, such as edema, erythema, burns, photoaging, and cancer, through altered intracellular signaling pathways associated with DNA damage, oxidative stress, inflammation, and cell cycle arrest. Therefore, ameliorating UVB-induced oxidative or inflammatory responses may be a potential strategy for prevention and protection against cellular and tissue damage. In this regard, a variety of edible and medicinal plants have been recently investigated for topical application. Natural substances have antioxidative and anti-inflammatory effects, depending on the type and ingredients, and they are generally rich in beneficial effects to humans. Therefore, naturally occurring chemicals derived from plants can be utilized as pharmaceutical or cosmetic ingredients for skin disease. The ACC used in this study is a natural mixture obtained by vacuum low–temperature distillation–extraction through an appropriate ratio of 14 natural substances, including *Phellodendron* bark, *Scutellaria baicalensis*, *Mentha arvensis* var. *piperascens*, and *Paeonia lactiflora* Pall as key ingredients. The aim of this study was to evaluate the protective effect of ACC on oxidative stress, inflammation, and apoptosis in human HaCaT keratinocytes.

Human skin has its own antioxidant mechanisms that can cope with the oxidative stress produced by UVB exposure. In other words, the network of intracellular antioxidative enzymes, which plays an important role in balancing the ROS levels in cells, is being used to maintain the normal state [24]. However, exposure to high-dose UVB or sustained UVB beyond the limits of the skin’s antioxidative mechanisms produces ROS that overwhelm antioxidative capacity and affect important cellular components, such as DNA, proteins, lipid membranes, and mitochondria. As a result, cutaneous antioxidants are depleted, and oxidative stress can lead to damage such as burns and precancerous growth [25,26,27]. A previous study has shown that exposing human or mouse skin to UVB radiation results in the production of excessive ROS beyond the antioxidative defense system [28]. Eventually, the occurrence of ROS in biological systems leads to DNA damage, various inflammatory disorders, and skin cancer. If the excessive generation of ROS is not rapidly eliminated, it can attack biological macromolecules such as DNA, proteins, and lipids, disrupting cellular function and resulting in extensive cellular oxidative damage that reaches apoptosis. Therefore, to provide a scientific basis for whether ACC contains skin protective ingredients, we focused on identifying the protective mechanism of ACC against UVB-induced oxidative stress in HaCaT cells. Prior to assessing the overall efficacy of ACC, we measured cell viability based on ACC concentrations and UVB doses in HaCaT cells. Under the experimental conditions, no significant cytotoxicity was observed up to a 100 µg/mL concentration of ACC, and the first significant cytotoxicity was observed at a 50 mJ/cm^2^ dose of UVB (Figure 3A,B). These two conditions were set as the reference point to be used in the experiments.

Minimal erythema dose (MED) refers to the amount of UV light needed to make the skin noticeably red in vivo. Reich et al. (2013) reported that the UVB dose causing apoptosis is very similar to the MED of UVB, and that UV-induced erythema is an inflammatory response caused by the appearance of sunburn cells with apoptosis occurring in human epidermis [29]. We conceived that MED is a dose that causes apoptosis and thought that if ACC can down-regulate pro-apoptotic molecules induced by UVB, it will be able to protect cells from UVB below a specific dose. The 50 mJ/cm^2^ UVB set in this study is the first dose that significantly decreases the viability of HaCaT cells in vitro, and it can be considered to correspond to MED. In conclusion, it was demonstrated that 100 µg/mL ACC pretreatment could protect HaCaT cells from cytotoxicity induced by 50 mJ/cm^2^ UVB. Of course, it cannot be concluded that the UVB conditions set in this study are consistent with the MED that will be applied to human body application tests using cosmetics containing ACC in the future, but it is considered to be helpful in presenting the criteria for acceptable UVB. Subsequently, DPPH free radical scavenging activity was measured to evaluate the antioxidative effect of ACC. Our results show that ACC appears to be somewhat weaker than ascorbic acid, quercetin, and baicalin, which have strong antioxidant capacity at relatively low concentrations. Although it is a roughly refined mixture rather than a single commercially available substance, it appears to be similar to the tendency of berberine and, given that, it represents a significant antioxidative effect at a 100 µg/mL concentration (Figure 4A). ACC shows that it can be a useful raw material to inhibit oxidative damage from ROS. We further confirmed that intracellular ROS, such as H_2_O_2_ and O_2_^–^, were noticeably increased at 1 day after UVB (50) irradiation in HaCaT cells and were effectively reduced by ACC pretreatment (Figure 4B,C). These results suggest that ACC has a clearly effective antioxidative effect against UVB irradiation in HaCaT cells.

The major changes in inflammation and immunoregulation following UVB exposure are the release of pro-inflammatory mediators and the expression of inflammatory enzymes. For example, TNF-α is a cytokine involved in the early stages of inflammation released when keratinocytes are damaged and amplifies the response by stimulating adjacent keratinocytes [30]. COX-2 is known as a rate-limiting enzyme that catalyzes the production of prostaglandin E2 (PGE2) from prostanoid precursors and can be stimulated by some cytokines in an inflammatory environment [31]. UVB irradiation also triggers the production of nitric oxide (NO) associated with the onset of various inflammatory skin diseases [32]. NO is produced by the enzymatic action of iNOS, which is strongly induced by environmental factors, microbial infections, or inflammatory cytokines in various cells [32,33,34]. Abnormal pro-inflammatory cytokine, COX-2, and iNOS overexpression patterns are induced by UVB irradiation resulting from the failure to regulate intracellular signaling pathways mediated by various transcription factors and upstream kinases. Unregulated inflammatory responses trigger noxious effects on cells and tissues through the production of excessive pro-inflammatory mediators and inflammatory enzymes, so controlling them can be a great help in protecting and preventing skin cells. In accordance with our experimental data, we confirmed that TNF-α and IL-1β, COX-2, and iNOS were markedly increased at 1 day after UVB irradiation in HaCaT cells and were effectively inhibited by ACC pretreatment (Figure 5). Therefore, we know that ACC has a clearly effective anti-inflammatory effect against UVB stress in HaCaT cells.

MAPKs are a large family of protein kinases that successively phosphorylate a series of separated cascades in response to various stimuli associated with the control of development, growth, differentiation, inflammation, and apoptosis [35]. JNK, p38, and ERK are each of the MAPK families present in mammalian cells, each forming a signaling pathway [36]. Accumulating evidence suggests that there are several studies in which UVB radiation activates MAPK signaling pathways either directly or indirectly through ROS-mediated pathways [37,38,39]. The expression of inflammatory cytokines has been shown to be affected by various intracellular signaling MAPK proteins, such as JNK, p38, and ERK [40,41]. UVB exposure induces the rapid activation of the p38 MAPK signaling pathway, leading to COX-2 expression in HaCaT cells [42]. In general, the JNK and p38 cascades are activated in response to cellular stress and are considered to perform both cell protection and antiapoptotic effects. On the other hand, the ERK cascade is activated by mitogenic stimulation and seems to mediate both cell proliferation and survival. In particular, the ERK signaling pathway is also known to include multiple signaling pathways used to perform a variety of functions [43,44]. However, recent studies suggest that the continuous activation of ERK is associated with the apoptotic process [45,46,47]. Several studies have reported that strong ERK activation inhibits the cell cycle through the induction of cell cycle inhibitory proteins, including p21Cip/Waf and p27KIP, and that ERK activation induced by DNA damage is related to the stimulation of apoptosis [48,49,50]. Exposure to UV radiation induces ROS production in skin cells, which activates intracellular signaling pathways that activate kinases such as JNK and p38. A previous study showed that acute UV radiation exposure activates three MAP kinases, JNK, p38, and ERK, within 1 h [51]. After all, lowering the abnormal state of MAPK activation to the basal level or keeping it close to the steady state may be an important approach to maintaining normal cell mechanisms from UV stress. Our observations showed that ACC pretreatment significantly reduced the excessive expression of p-SAPK/JNK, p-p38, and p-ERK1/2 in UVB-irradiated HaCaT cells. Additionally, ACC was confirmed to reduce the abnormal expression of p-ASK1, which is known as the upstream signal of MAPK (Figure 6). These findings suggest that ACC could control the aberrant activation of MAPK signals induced by UVB irradiation.

Until 1997, there were disagreements about whether HaCaT cells were appropriate for use in apoptosis studies due to the p53 mutations in HaCaT cells. It was known that p53 mutations in HaCaT cells, found at the dipyrimidine sites in codon 179 of exon 5 and codons 281 and 282 of exon 8, were related to the immortalization mechanism of this cell line, but it was not known whether the mutated p53 protein of HaCaT keratinocytes still functioned. Therefore, research on apoptosis using HaCaT cells was sometimes considered inappropriate to use this cell line because the apoptotic pathway would not function properly. However, Henseleit et al. (1997) showed that despite the mutations of the p53 gene in HaCaT cells, p53 can be involved in the UVB-induced apoptotic pathway in HaCaT keratinocytes [52]. After that, studies on apoptosis using UVB-induced HaCaT cells began to appear [53,54], and this study also attempted to evaluate whether ACC, the major substance, could impart an antiapoptotic effect to down-regulate pro-apoptotic molecules that begin to appear at a specific UVB dose.

UVB can induce apoptosis via the intrinsic pathway involving direct DNA damage, via the extrinsic pathway, including activated cell death receptors, or via ROS production [55,56,57]. Severe external stress induces excessive phosphorylated forms of MAPK modules, such as JNK, p38, and ERK, and it leads to apoptosis cascades through pro-apoptotic signals. Subsequently, bax makes numerous holes in the mitochondria, spilling the contents of the mitochondria into the cells [58]. In particular, caspase, an essential group of proteases required in the apoptotic process, is a key protein of both apoptotic pathways. Caspases are synthesized in zymogens, an inactive precursor from resting cells. Caspase-9 is an important factor in UV-induced apoptosis of keratinocytes and is involved in the intrinsic pathway, and caspase-8 is known as an initiator caspase via the extrinsic pathway [56]. Caspases are activated in cells through partial protein cleavage. The active form of cleaved caspases consequently results in the cleavage of PARP, and cleaved PARP performs apoptosis with cleaved caspase-3 [59]. In the case of skin, the exposure of skin cells to a manageable level of external stress induces proper apoptosis by their own protective mechanisms, but exposure to overwhelming UVB beyond the protective mechanisms can lead to necrosis or turn into tumorigenesis. Therefore, maintaining a normal apoptotic process is an issue that is directly related to skin health care. Based on these various facts, our study confirmed the activity of UVB-induced pro-apoptotic molecules and then demonstrated the antiapoptotic effect of ACC through experiments. Our results show that ACC pretreatment significantly reduced excessive expression levels of bax, c-caspase-9, c-caspase-3, c-caspase-8, and c-PARP in UVB-irradiated HaCaT cells (Figure 7). In other words, these results suggest that ACC could attenuate the pro-apoptotic signaling molecules induced by UVB irradiation and help maintain a basal level of apoptotic process. Taken together, we examined the mechanism of ACC in a series of signaling pathways leading to MAPK-mediated inflammation and apoptosis triggered in ROS production. Our findings represent a significant step forward in our understanding of ACC as a cosmeceutical material.

## 5. Conclusions

**Figure 8:**
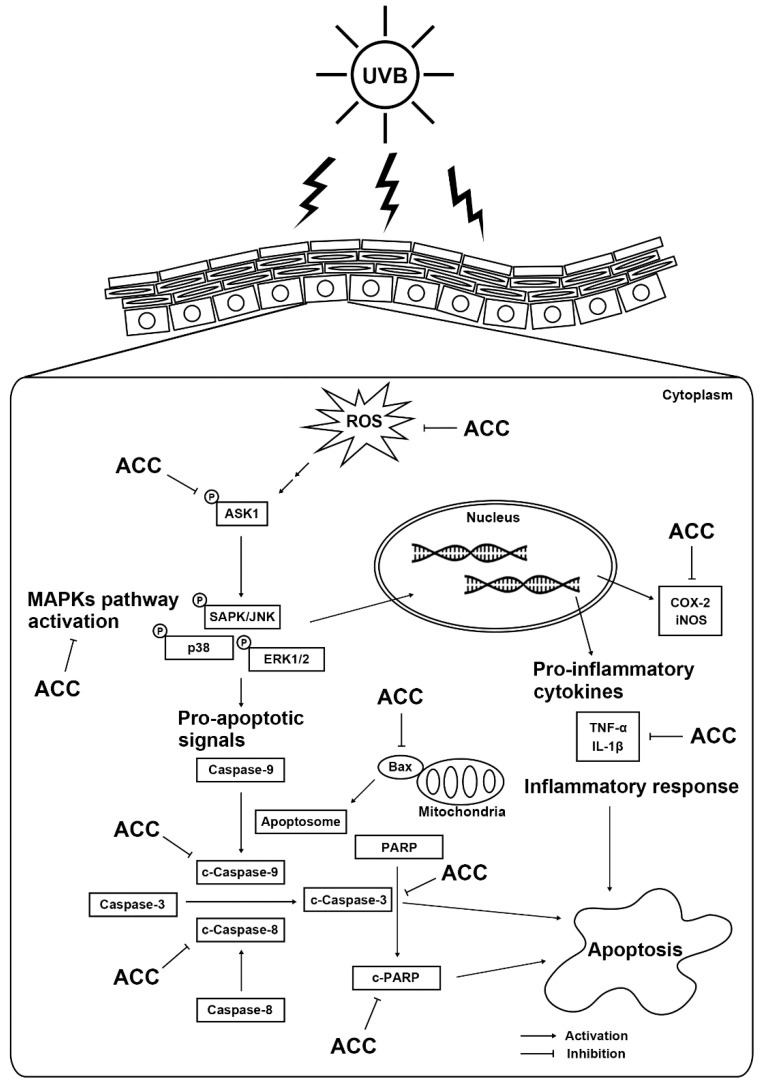
Scheme of the beneficial effects of ACC in UVB-irradiated HaCaT cells.

We demonstrated for the first time the beneficial effects of ACC on UVB-induced HaCaT cells. The proposed mechanism can be outlined as follows (Figure 8). ACC concentrations up to 100 µg/mL were not toxic and had a significant antioxidative effect through free radical scavenging. In addition, ACC has cytoprotective effects against UVB-induced cytotoxicity in HaCaT cells by inhibiting abnormal inflammation and apoptosis through the regulation of MAPK signals such as JNK, p38, and ERK. However, since this study validates the efficacy of ACC at the in vitro level, further studies are needed to elaborate on whether the desired effect is achieved in vivo to use ACC as a cosmeceutical raw material. Thus, we suggest that ACC is a natural mixture that includes beneficial ingredients against UVB stimulation and could offer potential agents for skin health.

## Figures and Tables

**Table 1:** Materials and proportion in ACC extracts.

Materials	Proportion (%)
*Phellodendron* bark	24
*Scutellaria baicalensis*	5
*Paeonia lactiflora* Pall.	12
*Dictamnus dasycarpus* Turcz.	12
*Anemarrhena asphodeloides*	6
Alumen	12
*Dryobalanops aromatica* Gaertner	2
*Mentha arvensis* var. *piperascens*	4
*Inula helenium*	3
*Syringa velutina* var. *kamibayashi*	1
*Corydalis incisa*	6
*Eclipta prostrata*	6
*Lonicera japonica*	1
*Glycyrrhiza uralensis*	6
